# Polyphenol-Rich Dry Common Beans (*Phaseolus vulgaris* L.) and Their Health Benefits

**DOI:** 10.3390/ijms18112331

**Published:** 2017-11-04

**Authors:** Kumar Ganesan, Baojun Xu

**Affiliations:** Food Science and Technology Program, Division of Science and Technology, Beijing Normal University—Hong Kong Baptist University United International College, Zhuhai 519087, China; kumarganesan@uic.edu.hk

**Keywords:** polyphenols, *Phaseolus vulgaris*, anti-oxidants, degenerative diseases, health-promoting effects

## Abstract

Polyphenols are plant metabolites with potent anti-oxidant properties, which help to reduce the effects of oxidative stress-induced dreaded diseases. The evidence demonstrated that dietary polyphenols are of emerging increasing scientific interest due to their role in the prevention of degenerative diseases in humans. Possible health beneficial effects of polyphenols are based on the human consumption and their bioavailability. Common beans (*Phaseolus vulgaris* L.) are a greater source of polyphenolic compounds with numerous health promoting properties. Polyphenol-rich dry common beans have potential effects on human health, and possess anti-oxidant, anti-diabetic, anti-obesity, anti-inflammatory and anti-mutagenic and anti-carcinogenic properties. Based on the studies, the current comprehensive review aims to provide up-to-date information on the nutritional compositions and health-promoting effect of polyphenol-rich common beans, which help to explore their therapeutic values for future clinical studies. Investigation of common beans and their impacts on human health were obtained from various library databases and electronic searches (Science Direct PubMed, and Google Scholar).

## 1. Introduction

Plants synthesize secondary metabolites that often have widespread bioactivities, and are known as phytochemicals. Polyphenol is one of the phytochemicals containing large bioactive structural phenolic units. It has a wide range of classification and possesses various pharmacological and health-promoting effects [1]. Polyphenols are largely found in fruits, cereals, vegetables, food legumes, herbs, spices, nuts, wine, olive oil, tea, coffee, and chocolate. Polyphenols are classified into different groups based on the function of several phenyl rings, including flavonoids (flavones, flavonols, flavanones, isoflavones, anthocyanins, chalcones, dihydrochalcones, and catechins), phenolic acids (hydroxybenzoic hydroxyphenyl acetic, hydroxyphenyl pentanoic and hydroxyl cinnamic acids), stilbenes, and lignans [2]. The primary functions of polyphenols are as anti-oxidants involved in the prevention of degenerative diseases such as cancer and metabolic syndromes [2]. The health-promoting effects of polyphenols depend on the quantity consumed in the diet and their bioavailability. In addition, polyphenols are the active substances in many food legumes, which regulate the activity of a broad spectrum of cell receptors, enzymes and gene expression [3]. Animal experimental studies showed that polyphenol in common beans possess anti-oxidant properties and have various biological activities including anti-diabetic, anti-obesity, anti-inflammatory, antimicrobial, anticancer, hepatoprotective, cardioprotective, nephroprotective, neuroprotective, and osteoprotective [4,5,6,7,8,9,10,11].

## 2. Common Beans and Their Health Benefits

Common beans (*Phaseolus vulgaris* L.) are annual plants, cultivated in temperate and semitropical regions for their edible dry seeds that are variously called navy beans, kidney beans, red beans, black beans, pinto beans, and cranberry beans. They were first cultivated in Peru and Mexico around 8000 years ago and are now cultivated worldwide [12]. They belong to the family Fabaceae. In the temperate regions, the green leaves and immature pods are edible as vegetables. Dry beans are mainly consumed in low- and middle-class families as the large portion of the protein. In many parts of Asian, young leaves are consumed as a salad. The straw of the plant is normally used for fodder after beans are harvested. In 2010, the global production of dried beans was 25.42 million metric tons, and they were harvested on 32 million hectares. About 19.23% of the productions was in India followed by Myanmar (13.88%), Brazil (12.42%), USA (5.66%), China (5.26%), Mexico (4.52%) and Tanzania (4.28%) [12]. The production of dried beans worldwide in 2014 was 27.59 million metric tons, and they were harvested on 31.22 million hectares. About 16.85% of the productions was in Myanmar followed by India (14.89%), Brazil (11.92%), USA (4.74%), Mexico (4.60%), Tanzania (4.023%) and China (3.84%) [12]. Beans are known to be used for treating eczema, diabetes, diuretic, burns, acne, cardiac, bladder, carminative, dropsy, dysentery, emollient, hiccups, itchy, and rheumatism [13].

Common beans do not differ mostly in their nutritional compositions; they differ slightly in taste, texture and cooking times [14]. Navy beans are white in color, and were used in the U.S. Navy diet during the 19th century; hence, their name. They are small-sized, white-skinned, oval-shaped beans. Navy bean-containing diets exerted beneficial effects during experimental colitis by reducing inflammatory biomarkers both locally and systemically [15]. Emerging evidence supports the efficacy of navy beans in regulating serum cholesterol and lipid profiles, and inhibiting the incidence and recurrence of adenomatous polyps or precancerous growths, thereby preventing colorectal cancer [16,17]. Kidney beans are large-sized, firm textured, red/pink glossy skinned and kidney-shaped beans. They have the potential to reduce glycemic index in experimental diabetes [18] and the ability to attenuate colonic inflammation in healthy mice [19]. Red beans are small, soft red textured, oval-shaped beans. They exert an anti-inflammatory response [20] and have health-promoting potential with anti-fungal, immunomodulatory, anti-proliferative and apoptosis-inducing activities in tumor cells [21,22]. Black beans are known as turtle beans, which are sweet in taste, soft texture, medium-sized, and oval-shaped beans. These coats are an excellent source of anthocyanins and other phenolics with the potential to be used as natural food colorants with exceptional anti-diabetic potential [23]. Pinto beans are medium-sized, brown-skinned, oval-shaped beans. Hemagglutinins, defensins isolated from pinto beans, possess anti-fungal, anti-diabetic and anti-tumor activities [24,25]. Cranberry beans, also called Roman beans, are red creamy textured, medium-sized and oval-shaped beans. They are rich in phenolic compounds and non-digestible fermentable components, which may help alleviate experimental colitis and mitigate the severity of other gut barrier-associated pathologies [26].

Common beans play a vital role in the vegetarian diets and provide numerous health benefits connected with eating pattern [27]. They serve as a cost-effective source of nutrients. Health benefits of beans are generally acquired from direct attributes, including their high content of proteins, dietary fibers, low saturated fat content, vitamins, minerals, and phytochemicals, as well as replacement in the diet, when they substitute for animal products [28]. These replacements of meat and other animal products with beans are highly linked with enhanced animal welfare and the decrease in inputs of environmental resources [28]. Sufficient amounts of polyphenols in the dried beans act as potent anti-oxidants. Regular intake of these dried beans containing total and soluble fiber as well as resistant starches have reduced glycemic index in the human. Studies have also suggested that diets that include beans reduce low density lipoprotein (LDL), increase high density lipoprotein (HDL) levels and positively affect risk factors for metabolic syndrome, and thereby decrease the risk of cardiovascular diseases (CVD) obesity and diabetes [29]. The Food Habits in Later Life study conducted in Japanese, Greek and Australian populations have demonstrated that dried beans and other food legumes are the only foods linked with a reduced risk of mortality [30]. Hence, health-promoting effects are directly proportional to increased bean intake.

## 3. Nutritional Compositions of Common Beans

Dry common beans (fully matured and dried) are a rich source of proteins, starch, unsaturated fatty acids (linoleic acid), dietary fibers, vitamins and minerals that are considered as important food resources. These dry beans are normally soaked and cooked for few hours, and served as soups, stews, and meat dishes. Green beans (green immature pods) have greater quantities of vitamin C and dietary fiber and are often sold as canned or frozen in the USA, while sold as fresh vegetables in China. Nutritional properties of the beans are highly linked to their measure of protein, and, to a smaller extent, their carbohydrate, vitamin, and mineral contents. The protein present in the bean types is different based on the cultivars, which ranges between 15% and 35%. The predominant amino acids present in the dry beans are lysine (6.5–7.5 g/100 g protein) and tyrosine with phenylalanine (5.0–8.0 g/100 g protein) [31]. Consequently, the protein present in the beans meets the minimal need of human requirements endorsed by the World Health Organization and Food and Agriculture Organization. Thus, 100 g of dry common beans serve in human provides about 9–25 g of protein, which is almost 20% of the recommended daily consumption for a normal adult. In addition, the digestibility of the dry bean protein is almost 80% [32]. About 55–75 g of carbohydrates are present in 100 g of raw beans and predominant fraction in the bean is starch, constituting almost 50% of the seed weight. In addition, dietary fibers (14–19 g/100 g of raw) and oligosaccharides are significant quantities [33]. More than 50% of fibers are insoluble, composed of pectins, pentosans, hemicellulose, cellulose, and lignin. The lipid fraction in the bean is about 1.5–6.5 g in 100 g of raw beans and is mainly composed of mono- and polyunsaturated fatty acids [34].

Dry beans contain biologically active phytochemicals, which are beneficial for human health [3]. While the beans contain huge quantities of protein, it is connected with anti-nutritional factors and other substances that are harmful to human health including polyphenols (including tannins), proteases, lectins, anti-vitamins, galacto-oligosaccharides, flatulence factors, allergens, and phytic acid [35]. Among the anti-nutritional factors, polyphenols are the primary contributors to reduce digestion of the bean in the human. They are highly active and can react with protein to cause impairment of the digestion. Tannins in the beans are potent and have the ability to bind with proteins by H-bonds, and thus prevent their digestion [36]. Boiling bean seeds is the common method of processing, and results in a decrease in the polyphenol content and reduces anti-nutritional factors [37]. The germination mechanism is also improving the levels of free amino acids, nutritional quality, and decreases the anti-nutritional factors [38]. The nutritional compositions of common beans are listed in Table 1.

Common beans have the highest source of protein and other dietary nutrition by complementing other foods, including meat, wheat, cereals and other food legumes [39]. The protein content of beans is almost equal to that of meat, ranging between 20% and 30% [40,41]. The primary protein fractions in beans are globulin (50–70%) and albumin (10%). Based on the sedimentation coefficient, the protein fractions of globulins are classified into 7S and 11S; both are natural oligomers [42,43]. The 7S fraction is normally referred to as phaseolin, an active glycoprotein consisting of about 50% of the total bean nitrogen, whereas the 11S globulin fraction is only 10% [44]. Prolamine and glutelin are also present as in minor quantities [45]. Common beans contain the highest ranges of glutelins (20–30%) when compared with other food legumes (7–15%) [46,47]. Similar to other food legumes, common beans contain a greater amount of essential amino acids, including lysine, which is deficient in most cereals. Beans are an excellent source of micronutrients such as minerals and vitamins and observed superior to cereals [48]. They have the highest vitamin and mineral contents comparing to all legumes [49]. Similar to other food legume seeds, dried beans contain numerous bioactive compounds such as galacto-oligosaccharides, protease inhibitors, lectins, phytates, oxalates and phenolic-rich substances that play crucial metabolic function in humans and animals [50]. Based on the diet quality, some of these substances have been known as anti-nutritional factors. These substances can reduce protein digestibility, diminish nutrient absorption and mineral bioavailability, which may cause flatulence in human [51]. However, these anti-nutritional factors have antioxidant and prebiotic activities, and protect DNA damage against various cancers [52,53,54,55,56]. Hence, common beans are nutritionally complementary with respect to essential amino acids, vitamins, minerals, and anti-nutritional factors, and the consumption of common beans could alleviate deficiency status, ensuring in the balanced diet [47,57].

## 4. Polyphenols in Common Beans

The dry bean contains plenty of polyphenols. Studies have demonstrated that phenolic compounds are predominantly located in the seed coat of the bean than in the cotyledon and testa [61]. The content of the phenolic compound is about 145 mg/g and represents about 11% of the total seed [62]. The phenolic compounds in the seeds are flavones, monomers, and oligomers of flavanols, flavanones, isoflavonoids, anthocyanins, chalcones, and dihydrochalcones [61,63,64,65,66]. However, the phenolic acids and non-flavonoid phenolic compounds (hydroxybenzoic and hydroxycinnamic acid) are mainly found in cotyledons of the bean [67]. Based on their chemical structure, they are a highly diverse group ranging from simple molecules such as phenolic acids to complex polymers such as tannins and lignin [68]. The testa of the beans contains greater quantities of proanthocyanidins and anthocyanins [61]. Condensed tannins (10.65 mg catechin equivalents/g) and cyanidin 3-glucoside (3.75 mg catechin equivalents/g) are also mainly present in seed coats of the bean [69,70]. These phenolic compounds are generally varied, based on the seed coat color pattern and types of the cultivar of the beans. The color of the seed coat is based on the presence of polyphenols including anthocyanins, flavonols glucosides, and condensed tannins. Dark-colored beans normally have the highest anthocyanins content [71]. In addition, red, black and pink-colored varieties confer color to the bean seed coat due to their anthocyanins. The colors of light yellow or pink spot of the seed coat are generally based on the presence of condensed tannins [70].

Phenolic compounds isolation and characterization were initiated at early 1960, and four anthocyanin pigments (delphinidin 3-glucoside, petunidin 3-glucoside, malvidin 3-glucoside and 3,5-diglucosides) were extracted from the seed coat of black violet beans [72]. Later, anthocyanins, flavonols, and tannins from the different varieties of kidney beans were isolated and characterized by many researchers [73,74,75,76]. Studies have further demonstrated that wild and weedy Mexican beans are rich in anthocyanins (delphinidin, delphinidin 3-glucoside, petunidin, petunidin 3-glucoside, cyanidin, malvidin, malvidin 3-glucoside, pelargonidin and peonidin), present in 62 Mexican wild-type varieties [77,78,79]. Raw and cooked beans contain predominant quantities of flavonoids including quercetin, myricetin, cynidine, procyanidin, naringenin, catechin, hesperetin and kaempferol [61]. The significant glucosides of the flavonoids are apigenin 7-*O*-glucoside, quercetin 3-*O*-glucoside, myricetin 3-*O*-glucoside, naringenin 7-*O*-glucoside, quercetin 4-*O*-galactoside and kaempferol 3-*O*-glucoside [80]. Variations in the flavonoids and its glucosides were observed in different bean varieties. Kaempferol and its 3-*O*-glucosides are primarily found in pinto beans; diglucosides of kaempferol and quercetin are found in dark red kidney beans; 3-*O*-glucosides of malvidin, petunidin, and delphinidin are present in black beans; quercetin 3-*O*-glucoside and its malonates are found in trace quantity in light red kidney beans; and kaempferol monoglucoside, kaempferol 3-*O*-glucoside, and kaempferol 3-*O*-xylosyl glucoside are found in Italian beans [71,81].

Lima et al. [82] and Guajardo-Flores et al. [80] have analyzed in Brazilian beans containing non-glycosylated forms of isoflavonoids including daidzein (0.0082–0.1291 mg/g) and genistein (0.0026–0.0097 mg/g). Among them, black type of beans showed the highest concentrations of isoflavonoids and daidzein was the predominant compound. Raw and cooked beans contain phenolic acids, which may be derived from benzoic acid (vanillic, *p*-hydroxybenzoic, and gallic acids) and those derived from cinnamic acid (ferulic, *p*-coumaric, and chlorogenic acids). Among them, ferulic acid is the predominant phenolic acid on the dry common beans [78]. Studies have demonstrated that cooking of common beans at high temperature does not change the content of phenolic acids [83]. Raw common beans contain *p*-hydroxybenzoic acid (0.0045–0.0086 mg/g), vanillic acid (0.0052–0.0166 mg/g), coumaric acid (0.0032–0.0068 mg/g), and ferulic acid (0.0017–0.0036 mg/g) [84]. The polyphenols present in the common beans are illustrated in Table 2.

## 5. Health Promoting Effects of Polyphenol-Rich Dry Beans

**Figure 1:**
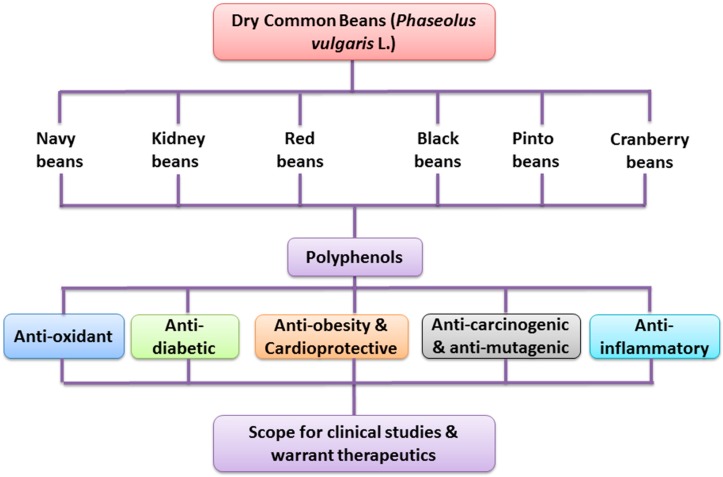
Health promoting effects of polyphenol-rich dry common beans.

The consumption of dry common bean has been greatly connected with many physiological and health promoting effects such as prevention of cardiovascular diseases, obesity, diabetes mellitus and cancers [86,88]. The anti-oxidant properties of polyphenol lie in their ability to neutralize free radicals and the chelation of transition metals, thus they counteract the initiation and propagation of oxidative processes [83]. Health promoting effects of polyphenol-rich dry common beans are illustrated in Figure 1.

### 5.1. Anti-Oxidant Activity

The dry common beans have excellent anti-oxidant activities because of its phenolic acids, flavonoids, stilbenes, and tannins. These anti-oxidant activities are primarily due to the reducing capacity of polyphenols as they play vital functions in neutralizing free radicals and scavenging radicals or suppressing lipid peroxidation [89]. In addition, polyphenols involve chelation of metal ions, causing impairment/cessation of oxidative mechanisms. Generally, the anti-oxidant activity is elevated during digestion and absorption of the common beans in the intestine. Normally, phenolic compounds are released higher in the stomach due to its acidic environment, and the acid medium and enzyme-mediated hydrolysis facilitate the higher solubility of polyphenols along with starch and proteins [65]. Common beans containing polyphenols have demonstrated the highest total anti-oxidant capacity measured by in vitro methods of 2,2’-Diphenyl-1-picrylhydrazyl (DPPH), β-carotene bleaching, ferric reducing anti-oxidant power, oxygen radical absorbing capacity, Trolox equivalent anti-oxidant capacity, and total radical-trapping anti-oxidant parameters [63,64,80,85,90,91,92,93]. Animal studies have also confirmed that common beans possess the highest anti-oxidant capacity, as measured in various biochemical parameters including thiobarbituric acid reactive substances (TBARS), hydroperoxides, glutathione (GSH), superoxide dismutase (SOD), catalase (CAT), glutathione reductase (GR), glutathione peroxidase (GPx), and glutathione S-transferase (GST) [26,61,93,94]. In vitro and in vivo studies of common beans exerting anti-oxidant activity are summarized in Table 3.

### 5.2. Anti-Diabetic Activity

Venn and Mann [150] have strongly suggested that the regular consumption of dry common beans is beneficial in the prevention and management of diabetes. Clinical studies show that consumption of three or more servings of beans in a week decreases the menace of diabetes almost by 35%, as compared to less or non-consumption of beans [151]. In vitro anti-diabetic studies of common beans have showed a greater inhibition of *α*-amylase, α-glucosidase and dipeptidyl peptidase-IV, which have found to be anti-hyperglycemic activities due to their phenolic compounds such as flavonoids and their glucosides of delphinidin, petunidin, and malvidin, anthocyanins, catechin, myricetin 3-*O*-arabinoside, epicatechin, vanillic acid, syringic acid, and *O*-coumaric acid [23,101,152,153]. In vivo studies have also demonstrated that beans containing phenolic compounds reduce blood glucose, glycosylated hemoglobin and elevated insulin levels in the animals [104,105,106,131]. Similarly, Roman-Ramos et al. [154] have demonstrated that anti-hyperglycemic effect of kidney beans in healthy rats revealed 21% reduction in the graph plot under glucose tolerance curve when compared with 16% of the standard diabetic drug. Epidemiological studies associated with Chinese population have shown that regular intakes of common beans are inversely connected with the risk of type-2 diabetes [155]. Gupta et al. [103] and Tang et al. [156] have also studied in 56 diabetic subjects based on traditional and Ayurvedic principles, which have revealed that the regular consumption of black bean for three months reduces plasma glucose and glycosylated hemoglobin. The results showed that black bean ameliorates type-2 diabetes, which is due to black beans containing total phenolic, tannins and anthocyanins. In vitro and in vivo studies of common beans exerting anti-diabetic activities are summarized in Table 3.

### 5.3. Anti-Obesity and Cardioprotective Activity

Metabolic syndrome is the set of metabolic conditions connected with the threat of cardiovascular diseases, increased triglycerides (TG), total cholesterol (TC), low density lipoprotein (LDL), very low density lipoprotein (VLDL), blood pressure (BP), and glucose as well as lower levels of HDL and central adiposity [157,158]. Regular intake of dry common beans has proven to be favorable for healthy subjects as well as obese individuals by decreasing serum TC and LDL and elevating HDL [159]. Epidemiological and clinical studies have demonstrated that consumption of common beans inversely connected with the risk of cardiovascular and coronary arterial diseases [158,160]. Further, these studies revealed that consumption of beans four or more times per week reduced the risks of coronary arterial diseases (22%) and cardiovascular diseases (11%): serum TC declines of about 1% decrease the risk of coronary heart disease by 2%, while serum LDL declines of about 1% reduce the risk of both diseases by about 1% [160]. Two weeks of regular consumption of baked beans by hypercholesterolemic individuals showed a significant reduction of TC (12%) and LDL (15%) [161]. Another clinical trial has also investigated on the hypercholesterolemic subjects with consumption of 275 g of navy beans for three weeks found a reduction of both serum TC, and LDL up to 24% [162]. Eight weeks of consumption of one cup serving baked beans by hypercholesterolemic individuals showed a marked reduction of TC (6%) and LDL (5%) [160]. Similarly, consumption of dried, cooked pinto beans (130 g) four times a week significantly decrease serum TC and LDL in healthy individuals, resulted in decreasing the risk of cardiovascular disease by 20% [158]. Azuki bean juice supplementation to young women also showed significantly decrease TC and TG levels and proved to be anti-hypercholesterolemic [163]. Similarly, consumption of one serving of cooked beans on regular basis inversely associated with the risk of myocardial infarction up to 38% [164].

In vivo anti-obesity and cardioprotective studies of common beans have showed a greater reduction of TC, TG, free fatty acids (FFA), phospholipids, and FA composition of total lipids, and was have found to have anti-hyperlipidemic activities due to their phenolic compounds such as quercetin, quercetin 3-*O*-glucoside, kaempferol, *p*-coumaric acid, ferulic acid, *p*-hydroxybenzoic acid, vanillic acid [116,165], orientin, isoorientin, rutin, myricetin-3-rhamnoside, hyperoside, isorhamnetin-3-*O*-glucoside, isoquercitrin, myricetin, luteolin, quercetin, luteolin-7-*O*-glucoside, kaempferol-glucuronide, kaempferol, isorhamnetin-3-*O*-glucoside, caffeine, hydroxycinnamic acid, and proanthocyanidins [141]. In vitro *and* in vivo studies of common beans exerting anti-obesity and cardiovascular activities are summarized in Table 3.

### 5.4. Anti-Mutagenic and Anti-Carcinogenic Activities

Normally, the generation of ROS and oxidative stress damage macromolecules such as lipid, protein RNA, and DNA, which may cause chronic degenerative diseases, including cancer [166,167]. However, the occurrence of cancer can be decreased by lifestyle and dietary habit changes. Studies have also suggested that diets rich in common beans reduce the greater risk of various cancers including colon, breast, and prostate [130,134,137]. A larger study conducted in 41 countries found that the consumption of common beans reduced the morbidity by cancers such as colon, breast, and prostate [168]. Further, studies have revealed that consumption of beans two or more times per week reduced the risks of colon cancer up to 47% [107], prostate cancer about 22% [169] and breast cancer about 67% [170]. In vivo studies have also suggested consumption of beans reduced risk of various cancers [132,133,134,135,136]. Hangen and Bennink [171] investigated diets fed with black beans in rats and observed the lower the incidence of total tumor (54%) and adenocarcinoma (75%). Similarly, the effect of navy beans decreased the occurrence of total tumor (59%) and adenocarcinoma (44%). The anti-carcinogenic and anti-mutagenic activities of beans are highly associated with the presence of phenolic compounds as well as other bioactive compounds [62,93]. Phenolic compounds have the potential to inhibit mutagenic agents including polycyclic aromatic hydrocarbons, nitrosamines, and mycotoxins by inhibiting activation enzymes, provoking detoxification enzymes and intonation of commencement of mutagens [172,173]. Furthermore, common beans possess anti-carcinogenic and anti-mutagenic properties due to their phenolic compounds interacting with ultimate toxicants or mutagens, scavenging activities of phenolics, and inhibition of metabolism of the ultimate mutagen [62,76,174]. In vitro and in vivo studies on chemopreventive and anti-mutagenic activities of common beans are summarized in Table 3.

### 5.5. Anti-Inflammatory Activity

Common beans contain phenolic compounds (phenolic acids, flavonoids, and anthocyanins) and non-digestible fermentable components (short-chain fatty acid precursors) with demonstrated anti-oxidant and anti-inflammatory activities. Experimental studies associated with modulation of inflammatory-related cell signaling pathways by common beans have been well established. In an animal study, C57BL/6 mice fed a 20% navy bean or black bean flour-containing diet showed significantly reduced dextran sodium sulfate induced experimental colitis and inflammation-related parameters (IL-1β, TNFα, IFNγ, IL-17A, and IL-9), increased histological injury score and apoptosis, and alleviated symptoms of colitis and colon inflammation [15]. Common beans possess various bioactive compounds including flavonoids and anthocyanins, which significantly reduced the activity of murine macrophages through the inhibition of pro-inflammatory gene expression without cytotoxicity [117,118,119]. Similarly, human-randomized, controlled, crossover trials have also demonstrated that three-day intake of 100 g of black bean meal and soup improved the arthritic condition by significantly reducing pain and inflammation [119]. The immunomodulatory effects of 20% navy bean or black bean or cranberry bean administration in C57BL/6 mice for two weeks showed a significant reduction in colonic mucosal damage and inflammation in response to dextran sodium sulfate. The results further demonstrated that common bean containing bioactive compounds including phenolic acids, flavonoids, and anthocyanins provoke prominent immune response [26,120]. In vitro and in vivo studies of common beans exerting anti-inflammatory activities are summarized in Table 3.

## 6. Conclusions

Dry common beans are consumed in diets worldwide, and play a significant function in human nutrition, especially as a source rich in proteins, carbohydrates, dietary fibers, vitamins, minerals, phytochemicals and other micronutrients, as well as low saturated fat content. Besides these nutrients, common beans possess enormous quantities of polyphenols and other metabolites, have anti-oxidant activities, major role in health-promoting effects, and protect against various diseases including diabetes, CVD, cancer, and microbial infections. These health benefits of beans are generally acquired from direct attributes, including their high content of nutrients, as well as replacement in the diet, when they substitute for animal products. It can therefore be concluded that health-promoting effects are directly proportional to the increase in bean intake. In addition, long-term clinical studies are urgently needed to warrant the therapeutic benefit of polyphenols rich common beans. In addition, the synergistic effects of polyphenol-rich common bean with other bioactive compounds on biological functions would be a recommendation for further studies. This investigation proves the efficacy of bioactive compounds in common bean, and enhances the therapeutic options for various diseases.

## Figures and Tables

**Table 1:** Nutritional compositions of common beans in 100 g of edible portion [58,59,60].

Nutrient	Units	Navy Beans	Kidney Beans	Red Beans	Black Beans	Pinto Beans	Cranberry Beans
Energy	kcal	92	92	167	464	500	257
Protein	g	6.15	5.38	22.22	14.29	10.71	22.86
Total lipid (fat)	g	0.00	0.38	0.00	21.43	21.43	0.00
Carbohydrate	g	16.15	20.77	63.89	57.14	60.71	60.00
Total dietary fiber	g	4.6	5.4	44.4	14.3	10.7	25.7
Total sugars	g	0.00	0.77	2.78	3.57	3.57	2.86
Resistant starch	g	4.2	2.0	3.8	1.7	1.9	2.6
**Minerals**							
Calcium	mg	62	46	167	143	71	114
Iron	mg	1.38	1.38	7.29	3.86	2.57	5.1
Potassium	mg	300	268	222	279	96	265
Magnesium	mg	48	37	44	60	43	39
Sodium	mg	108	8	69	286	286	10
**Vitamins**							
Vitamin C	mg	0.9	0.9	0.0	0.0	0.0	0.0
Folate	µg	127	115	140	128	147	124
**Lipids**							
Total saturated	g	0.000	0.000	0.000	1.790	1.790	0.000
Total monounsaturated fatty acids	g	0.000	0.000	0.000	14.290	14.290	0.000
Total polyunsaturated fatty acids	g	0.000	0.000	0.000	5.360	5.360	0.000
Polyphenol	mg of gallic acid equiv/g	12.47	14.14	13.68	12.60	12.52	11.73
Flavonoids	mg of rutin equiv/g	1.78	2.59	1.55	1.28	0.98	1.65

**Table 2:** List of polyphenols in the common beans.

Bean Name	Polyphenol Class	Polyphenol Sub-Class	Compound Name	References
Dark bean	Flavonoids	Anthocyanins	Cyanidin 3-*O*-glucoside, pelargonidin 3-*O*-glucoside, petunidin-3-*O*-*β*-glucopyranoside, malvidin 3-*O*-glucoside, delphinidin acetyl-glucoside, pelargonidin acetyl glucoside, pelargonidin 3-*O*-malonyl glucoside, petunidin feruloyl glucose	[61]
Wild and weedy Mexican bean, pinto and black beans	Flavonoids	Anthocyanins	Peonidin, pelargonidin, cyanidin	[78,85]
Dark bean, Wild, and weedy Mexican bean	Flavonoids	Anthocyanins	Delphinidin 3-*O*-glucoside	[61,78]
Alubia, black, cranberry, dark red kidney, great northern, light red kidney, navy, pink, pinto, and small red	Flavonoids	Anthocyanins	Petunidin 3-*O*-(6″-acetyl-glucoside)	[71,81]
Dark and kidney bean, zolfino landraces	Flavonoids	Anthocyanins	Pelargonidin 3,5-*O*-diglucoside	[45,61]
Alubia, black, cranberry, dark red kidney, great northern, light red kidney, navy, pink, pinto, and small red	Flavonoids	Anthocyanins	Delphinidin 3-*O*-glucosyl-glucoside	[71,86]
Dark bean	Flavonoids	Flavanols	(+)-Catechin, (-)-epicatechin, (+)-gallocatechin, procyanidin dimer, (-)-epigallocatechin, Procyanidin dimer B2, procyanidin dimer B3, procyanidin dimer B4, procyanidin trimer, procyanidin trimer EEC, naringenin 7-glucoside	[61]
Dark bean	Flavonoids	Flavanones	Naringenin, hesperetin, naringin, naringenin 7-*O*-rutinoside, naringenin 7-*O*-glucoside, naringenin-7-methyl ether 2, hesperetin 3′-*O*-glucuronide, hesperetin 7-*O*-glucuronide, hesperetin 3′,7-*O*-diglucuronide, hesperetin 5,7-*O*-diglucuronide, hesperetin 7-*O*-rutinoside	[61]
Dark bean	Flavonoids	Flavones	Apigenin, apigenin 7-*O*-glucoside	[61]
Brazilian bean	Flavonoids	Flavones	Chrysin	[66]
Dark bean, Brazilian bean, Mexican bean	Flavonoids	Flavonols	Kaempferol	[35,61,66]
Dark bean, Brazilian bean, Mexican bean	Flavonoids	Flavonols	Quercetin	[35,61,66]
Dark bean, and Brazilian bean	Flavonoids	Flavonols	Quercetin 3-*O*-galactoside, Quercetin 3-*O*-glucoside, Quercetin 3-*O*-rutinoside, Myricetin, Myricetin 3-*O*-glucoside, Myricetin 3-*O*-rhamnoside, Kaempferol 3-*O*-glucoside, Kaempferol 3-*O*-rutinoside	[61,66]
Pinto beans, zolfino landraces	Flavonoids	Flavonols	Kaempferol 3-*O*-glucosylxylose	[63,87]
Alubia, black, cranberry, dark red kidney, great northern, light red kidney, navy, pink, pinto, and small red	Flavonoids	Flavonols	Kaempferol 3-*O*-xylosyl-glucoside	[71,81]
Pinto beans	Flavonoids	Flavonols	Kaempferol 3-*O*-acetyl-glucoside	[63]
Dark bean, Brazilian bean	Flavonoids	Isoflavonoids	Daidzein	[61,66]
Dark bean, Brazilian bean	Flavonoids	Isoflavonoids	Genistein	[61,66]
Dark bean	Flavonoids	Isoflavonoids	Biochanin A	[61]
Pinto and black beans	Flavonoids	Isoflavonoids	Glycitein	[85]
Dark bean	Flavonoids	Isoflavonoids	Dihydrogenistein	[61]
Brazilian bean	Polyphenols	Polyphenols	Coumestrol	[66]
Dark bean, pinto and black beans, Mexican bean	Phenolic acids	Hydroxybenzoic acids	Protocatechuic acid	[35,61,85]
Dark bean	Phenolic acids	Hydroxybenzoic acids	Gallic acid	[61]
Mexican bean	Phenolic acids	Hydroxybenzoic acids	Vanillic acid	[35]
Dark bean, Mexican bean	Phenolic acids	Hydroxycinnamic acids	*p*-Coumaric acid	[35,61]
Pinto and black beans	Phenolic acids	Hydroxycinnamic acids	Caffeic acid	[85]
Dark bean, Mexican bean	Phenolic acids	Hydroxycinnamic acids	Ferulic acid	[35,61]
Dark bean	Phenolic acids	Hydroxycinnamic acids	Sinapic acid, Ferulic acid 4-glucoside	[61]
Dark bean	Stilbenes	Stilbenes	*trans*-Resveratrol, resveratrol 3-*O*-glucoside	[61]

**Table 3:** Summary of *in vitro, in vivo* and clinical studies on health-promoting effects of polyphenol-rich common beans (*Phaseolus vulgaris* L.).

Bean Name	Polyphenols Names	Model/Subjects	Dosage	Experimental Period	Activities	References
Kidney bean	*p*-coumaric, ferulic and sinapic acids, quercetin, kaempferol, procyanidins B-2 and B-3 and tannins	*Brochothrix thermosphacta*, *Staphylococcus aureus*, *Listeria monocytogenes Scott A*, *Salmonella typhimurium*, *E. coli O157: H7*, *Pseudomonas fragi*, and *Lactobacillusplantarum*	62.5 to 500 µg/mL	36–48 h	Anti-bacterial activity	[95]
Perla black bean	Delphinidin 3-*O*-glucoside, petunidin 3-*O*-glucoside and malvidin 3-*O*-glucoside	Bacterial strain: *Pseudomonas aeruginosa*, *Proteus vulgaris*, *Klebsiella oxytoca*, *Enterococcus faecalis*, *Staphylococcus aureus*, *Staphylococcus epidermidis and Listeria monocytogenes*; Parasitic strain: *Giardia lamblia*, *Entamoeba hystolitica and Trichomonas vaginalis*	0.05, 0.5 and 5.0 mg/disk	36–48 h	Anti-bacterial and anti-parasitic activity	[96]
Peruvian and Brazilian bean	*Chlorogenic and caffeic acid*	In vitro	50 µL	24 h	Anti-diabetic and anti-hypertensive activity	[97]
Brown bean	Total phenolics	Human (16)-randomized crossover design	100 g/bw/p.o.	30 days	Anti-diabetic and anti-obesity activity	[98]
Kidney bean	Total phenolics and anthocyanins	Human with overweight subjects (39)-A randomized, double-blind, placebo-controlled clinical trial	50 g/bw/p.o.	60 days	Anti-diabetic and anti-obesity activity	[99]
Navy bean	Total phenolics and anthocyanins	3T3-L1 adipocytes	50 g/bw/p.o.	60 days	Anti-diabetic and anti-obesity activity	[100]
Pinto bean	Delphinidin glucoside, petunidin glucoside, malvidin glucoside, anthocyanins, catechin, myricetin 3-*O*-arabinoside, epicatechin, vanillic acid, syringic acid and *O*-coumaric acid	in vitro	50 µL	24 h	Anti-diabetic activity	[101]
Kidney bean	Phenolic acids and bioactive peptide fractions	In vitro	<1, 1–3.5, 3.5–5, 5–10, and 10 kDa	24 h	Anti-diabetic activity	[102]
Black bean	Phenolic acids and bioactive peptide fractions	In vitro	10.20 to 0.34 mg	24 h	Anti-diabetic activity	[23]
Black bean	Delphinidin-3-*O*-glucoside, petunidin-3-*O*-glucoside, and malvidin-3-*O*-glucoside	Caco-2 cells	Anthocyanin solutions (1 mg/mL), purified anthocyanins (100 µM malvidin, 100 µM delphinidin) or phloretin (100 µM).	24–36 h	Anti-diabetic activity	[23]
Black bean	Total phenolic, tannins and anthocyanins	Human (56)-diabetic patients	100 g of black bean	3 months	Anti-diabetic activity	[103]
Kidney bean	Total phenolic, tannins and anthocyanins	Wistar albino rats	200 mg/kg bw	30 days	Anti-diabetic activity	[104]
Black bean	Total phenolic, tannins and anthocyanins	Wistar albino rats	200 mg/kg bw	45 days	Anti-diabetic activity	[105]
Pinto bean	Total phenolics	Human (12)-randomized, double-blind, placebo-controlled study	100 g/bw/p.o.	3 h	Anti-diabetic activity	[106]
Navy bean	Total phenolics	Human with diabetes (17)-randomized 4 × 4 crossover trial	50 g/bw/p.o.	24 h	Anti-diabetic activity	[107]
Black bean	Total phenolics and anthocyanins	In vitro	100 µg	24 h	Anti-diabetic activity	[108]
White kidney bean	Total phenolics	Wistar albino rats	50 mg/kg bw/p.o.	7 days	Anti-diabetic activity	[109,110]
Black bean	Phenolic acids and bioactive peptide fractions	In vitro	10.20–0.34 mg	24 h	Anti-diabetic, and anti-hypertensive activities	[101]
Zolfino landrace	Phenolic acids	in vitro	700 µL	24 h	Anti-diabetic, anti-oxidant and anti-inflammatory activities	[111]
Kidney bean	Phenolic acids (chlorogenic acid, gallic acid, *p*-hydroxy benzoic acid, caffeic acid, protocatechuic acid, *p*-coumaric acid, rosmarinic acid, ferulic acid, sinapic acid and ellagic acid) and flavonoids (epicatechin, cate chin, gallocatechin gallate, epigallocatechin gallate, quercetin, hesperidin, and rutin)	Male Wistar rats	0.4, 0.8 and 1.2 g/kg bw/p.o. for 6 weeks	21 days	Anti-diabetic, hypolipidemic and cardioprotective activity	[112]
Kidney bean	Lectins and polyphenol	*Fusarium oxysporum*, *Coprinus comatus*, and *Rhizoctonia solani*	20–200 µg/mL	24 h	Antifungal activity	[113]
Kidney bean	Total phenolics	Sprague-Dawley rats	0, 7.5%, 15%, 30% or 60% *w*/*w*	7 days	Anti-hepatotoxic effect	[114]
White and red bean	Ferulic, coumaric, Sinapic acid, Catechin, Malvidin 6-*O*-glucoside, Quercetin,	Macrophages cell line RAW 264.7	20 µL	36–48 h	Anti-inflammatory activity	[115]
Navy and pinto bean	Phenolic acids and bioactive peptide fractions	RAW 264.7 macrophages	1–3.5, 3.5–5, 5–10, and 10 kDa	36–48 h	Anti-inflammatory activity	[102]
Black, navy, kidney and pinto bean	(+)-catechin	*Salmonella typhimurium* strains TA98 and TA100	2.5, 5, 10, 12.5, 15 and 25 µg	24 h	Anti-mutagenic activity	[62]
Black and kidney beans	Quercetin, kaempferol, *p*-coumaric acid, ferulic acid, *p*-hydroxybenzoic acid, and vanillic acid	Sprague-Dawley rats and a diet-induced obesity model in C57Bl/6 mice	7.5%, 15%, 30% or 60% *w*/*w*	7 days	Anti-obesity activity	[116]
Black bean	Total phenolics	In vitro	50–200 µL	24 h	Anti-oxidant and anti-inflammatory activities	[117]
Kidney bean	Flavonoids	HMEC-1 line	0.7 mg	36–48 h	Anti-oxidant and anti-inflammatory activities	[118]
Black bean	Total phenolic, tannins and anthocyanins	Human (12)-randomized, controlled, crossover trial	100 g of black bean meal and soup	3 days	Anti-oxidant and anti-inflammatory activities	[119]
Navy and black bean	Phenolic acids, flavonoids, and anthocyanins	C57BL/6 mice	20% navy bean or black bean/p.o.	2 weeks	Anti-oxidant and anti-inflammatory activities	[15]
White and dark kidney beans	Phenolic acids, flavonoids, and anthocyanins	C57BL/6 mice	20% navy bean or black bean/p.o.	2 weeks	Anti-oxidant and anti-inflammatory activities	[19]
Cranberry bean	Phenolic acids, flavonoids, and anthocyanins	C57BL/6 mice	20% navy bean or black bean/p.o.	2 weeks	Anti-oxidant and anti-inflammatory activities	[26]
Navy and black beans	Phenolic acids, flavonoids, and anthocyanins	C57BL/6 mice	20% navy bean or black bean/p.o.	2 weeks	Anti-oxidant and anti-inflammatory activities	[120]
Pinto, navy and black beans	(+)-catechin	*Salmonella typhimurium* strains TA98 and TA100	2.5, 5, 10, 12.5, 15 and 25 µg	24 h	Anti-oxidant and anti-mutagenecity activities	[90]
Pinto, navy and black beans	Phenolic acids and Lectin-free fractions	Human erythrocytes and *Saccharomyces cerevisiae* cells	0.2 mg	24 h	Anti-oxidant and anti-mutagenic effects	[93]
Black and kidney beans	Catechin	In vitro	50–100 µL	24 h	Anti-oxidant and anti-mutagenic activities	[91]
12 varieties of non-pigmented bean, red bean, speckled bean, and dark bean	Gallic acid, chlorogenic acid, epicatechin, myricetin, formononetin, caffeic acid, and kaempferol	In vitro human epithelial colorectal adenocarcinoma (Caco-2) cells, breast cancer (MCF-7), and A549 NSCLC cell line	15–300 µL	36–48 h	Anti-oxidant and anti-proliferative Activities	[121]
Black bean	Genistein, non-glycosylated flavonols	In vitro mammary gland, hepatic and colon cancer cell lines	50–200 µL	36–48 h	Anti-oxidant and anti-proliferative activities	[122]
Black bean	Total phenolics	Wistar albino rats	200 mg/kg bw/p.o.	45 days-	Anti-oxidant anti-diabetic and anti-hyperlipidemic activities	[123]
Dalia bean	Coumaric, salicylic, gallic, caffeic acids, epigallocatechin, rutin and quercetin, and flavonoids	In vitro	100 µL	24 h	Anti-oxidant activity	[124]
Brazilian bean	Ferulic, sinapic, chlorogenic, and hydroxycinnamic acids	In vitro	50 µL	24 h	Anti-oxidant activity	[125]
Pinto and black beans	Total phenolics, phenolic acids, isoflavones, and anthocyanins	In vitro	50 µL	24 h	Anti-oxidant activity	[64,85,91]
Brazilian bean	Total phenolics, and phenolic acids	In vitro	50–100 µL	24 h	Anti-oxidant activity	[126]
Black bean	Total phenolics	In vitro	200 µL	24 h	Anti-oxidant activity	[127,128]
Yellow string bean	Total polyphenolics	In vitro	10–100 µL	24 h	Anti-oxidant activity	[92]
Black bean	Total phenolics	Wistar albino rats	200 mg/kg bw/p.o.	45 days	Anti-oxidant activity	[94]
Black bean	(+)-catechin, quercetin, vanillin and ellagic, caffeic, ferulic, gallic, chlorogenic, and sinapic acids	Human and in vitro	1 g/p.o.	36–48 h	Anti-oxidant activity and enhance gastrointestinal digestion and simulated colonic fermentation	[129]
Dark bean	*p*-coumaric, ferulic, sinapic acids, myricetin, quercetin, kaempferol, flavanones, hesperetin and naringenin derivatives	In vitro cell line cultures of Astrocytes (U-373), renal adenocarcinoma (TK-10), breast adenocarcinoma (MCF-7) and melanoma (UACC-62)	700 µL	36–48 h	Anti-oxidant, neuroprotective and anticancer activities	[61]
Kidney bean	Total phenolics	Female Sprague Dawley rats	0, 7.5%, 15%, 30% or 60% *w*/*w*	46 days	Chemoprotective effect on breast cancer	[130]
Black bean	Total phenolics	Female Sprague Dawley rats	7.5%, 15%, 30% or 60% *w*/*w*	46 days	Chemoprotective effect on breast cancer	[131]
Black, pinto and kidney beans	Tannins	Male Sprague–Dawley rats	7.5%, 15%, 30% or 60% *w*/*w*	46 days	Chemoprotective effect on breast cancer	[132]
Black bean	Tannins	Male Sprague–Dawley rats	2.5 g/kg bw/p.o.	9 weeks	Chemoprotective effect on colon cancer	[133]
Black and navy beans	Tannins	Male Sprague–Dawley rats	2.5 g/kg bw/p.o.	9 weeks	Chemoprotective effect on colon cancer	[134]
Black beans	Tannins	Male Sprague–Dawley rats	2.5 g/kg bw/p.o.	9 weeks	Chemoprotective effect on colon cancer	[135,136]
Kidney bean	Tannins	Human HT-29 cell lines	100 µL	48 h	Chemoprotective effect on colon cancer	[137]
Kidney bean	Tannins	Sprague–Dawley rats and *Clostridium butyricum* strain MIYAIRI588	2.5 g/kg bw/p.o.	9 weeks	Chemoprotective effect on colon cancer	[138]
Black bean	(+)-catechin	Human HT-29 cell lines	20 mg	48 h	Chemoprotective effect on colon cancer	[139]
Black bean	Flavonoids	In vitro	5 mg	24 h	Cholesterol-lowering effects	[140]
Black bean	Orientin, isoorientin, rutin myricetin-3-*O*-rhamnoside, isorhamnetin-3-*O*-glucoside, isoquercitrin, myricetin, luteolin, quercetin, kaempferol, hyperoside, luteolin-7-*O*-glucoside, kaempferol-glucuronide, caffeine, isorhamnetin-3-*O*-glucoside, hydroxycinnamic acid, and proanthocyanidins	Male Wistar rats	2% bean seed coat extract/p.o.	7 days	Hypoglycemic and hypolipidemic effects	[141]
Black bean	Total phenolics	Wistar albino rats	200 mg/kg bw/p.o.	45 days	Hypoglycemic and hypolipidemic effects	[142]
Black bean	Flavonoids	Male Wistar rats	200, 400 mg/kg bw	7 days	Hypoglycemic effect	[143,144]
Navy and pinto beans	Flavonoids	Male Wistar rats	50, 200, 500 mg/kg bw	15 days	Hypoglycemic effect and anti-obesity effect	[145]
Kidney bean	Flavonoids	Male Wistar rats	50, 200, 500 mg/kg bw	21 days	Hypoglycemic effect and anti-obesity effect	[146]
Black bean	Flavonoids	Obese Zucker fa/fa rats	50 and 500 mg/kg bw	3–7 days, 20 days	Hypoglycemic effect and anti-obesity effect	[147]
Black bean	Catechin	Male Wistar rats	50 mg/kg bw	21 days	Hypoglycemic effect and anti-obesity effect	[109]
Black bean	Flavonoids	CD1 mice	200, 400 mg/kg bw	45 days	Hypoglycemic effect and anti-obesity effect	[148]
Black bean	Total phenolic, tannins and anthocyanins	CD1 mice	200, 400 mg/kg bw	21 days	Hypoglycemic effect and anti-obesity effect	[149]
Black bean	Quercetin 3-*O*-glucoside	Rat hepatocytes and C57BL/6 mice	25 mg	48 h	Hypolipidemic activity	[140]

Expansion: b.w- body weight; *w*/*w*- weight/weight; p.o.- per oral.

## Abbreviations

b.w.	Body weight
BP	Blood pressure
CAT	Catalase
DPPH	2,2’-Diphenyl-1-picrylhydrazyl
FA	Fatty acids
FFA	Free fatty acids
GR	Glutathione reductase
GSH	Reduced glutathione
GST	Glutathione-s-transferase
HDL	High density lipoprotein
IL	Interleukin
i.p.	Intraperitoneal
i.v.	Intravenous
kDa	Kilo daltons
LDL	Low density lipoprotein
MTT	3-(4,5-Dimethylthiazol-2-Yl)-2,5-diphenyltetrazolium bromide
p.o.	Per oral
ROS	Reactive oxygen species
SOD	Super oxide dismutase
TBARS	Thiobarbituric acid reactive substances
TC	Total cholesterol
TG	Triglycerides
TNF-α	Tumour necrosis factor α
VLDL	Very low density lipoprotein
*w*/*w*	Weight/weight
